# Marine Compounds for Melanoma Treatment and Prevention

**DOI:** 10.3390/ijms231810284

**Published:** 2022-09-07

**Authors:** Eleonora Montuori, Anita Capalbo, Chiara Lauritano

**Affiliations:** 1 Department of Chemical, Biological, Pharmaceutical and Environmental Sciences, University of Messina, Viale F. Stagno d’Alcontres 31, 98166 Messina, Italy; 2 Department of Ecosustainable Marine Biotechnology, Stazione Zoologica Anton Dohrn, Via Acton 55, 80133 Napoli, Italy

**Keywords:** microalgae, anti-cancer, melanoma, marine biotechnology

## Abstract

Melanoma is considered a multifactorial disease etiologically divided into melanomas related to sun exposure and those that are not, but also based on their mutational signatures, anatomic site, and epidemiology. The incidence of melanoma skin cancer has been increasing over the past decades with 132,000 cases occurring globally each year. Marine organisms have been shown to be an excellent source of natural compounds with possible bioactivities for human health applications. In this review, we report marine compounds from micro- and macro-organisms with activities in vitro and in vivo against melanoma, including the compound Marizomib, isolated from a marine bacterium, currently in phase III clinical trials for melanoma. When available, we also report active concentrations, cellular targets and mechanisms of action of the mentioned molecules. In addition, compounds used for UV protection and melanoma prevention from marine sources are discussed. This paper gives an overview of promising marine molecules which can be studied more deeply before clinical trials in the near future.

## 1. Introduction

**Figure 1:**
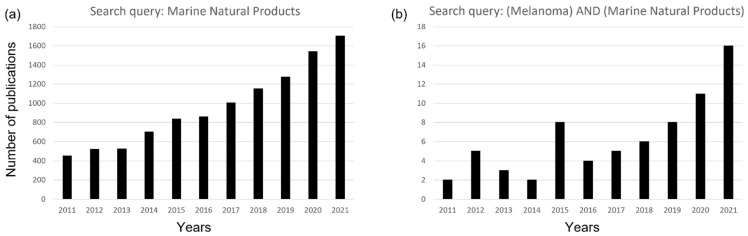
PubMed search results 2011–2021 by using as filters (**a**) the words “marine natural products” and (**b**) “melanoma“ and “marine natural products” in “all fields” query box.

Around 70% of the planet’s surface is covered by water [1] and marine environments have been shown to be characterized by a huge biological and chemical diversity. Over the past 50 years, approximately 38,662 marine natural products (MNPs) have been reported from marine species (https://marinlit.rsc.org/; accessed on 19 May 2022). Considering the increasing number of human diseases and antibiotic resistant infections, the scientific community has moved its attention to marine biodiversity to find new potential drugs. This interest is confirmed by the increasing numbers of scientific publications on marine natural products. Looking for “marine natural products” in the public database PubMed, there are 13,073 resulting publications, with an increasing trend over the years (Figure 1a). Looking for “melanoma” and “marine natural products” in the public database PubMed, the same trend is observed (accessed on 14 May 2022; Figure 1b).

According to the World Health Organization (WHO), the incidence of melanoma skin cancer has been increasing over the past decades with 132,000 cases occurring globally each year (https://www.who.int/news-room/questions-and-answers/item/radiation-ultraviolet-(uv)-radiation-and-skin-cancer; accessed on 16 May 2022). More solar UV radiation is reaching the Earth’s surface due to ozone level depletion, and the WHO reports an estimation of an additional 300,000 non-melanoma and 4500 melanoma skin cancer cases for each 10% decrease in ozone levels, resulting in a health and socio-economic problem [2].

A study published in 2020 in the International Journal of Cancer reported that 91% of all melanomas in United States and 97% in Hawaii were dependent on UV radiation, and first of all the sun radiation. Melanomas are also caused by genetic predisposition ad other phenotypic factors such as fair skin and many moles [3]. Another study published in 2020 in *Nature Genetics*, based on 37,000 melanoma cases in different world populations, demonstrated that there was an interaction of genetic predisposition and UV ray damage [4] (https://www.airc.it/cancro/prevenzione-tumore/il-sole/rischi-del-sole, accessed on 1 August 2022).

As reported by the National Cancer Institute (https://seer.cancer.gov/statfacts/html/melan.html; accessed on 16 May 2022), estimated new cases in 2022 are 99,780, with 7650 estimated deaths. According to the “Melanoma Tumors” section of the 4th edition of the WHO classification of skin tumors [5,6], melanomas are divided into those related to sun exposure and those that are not. As for sun-related melanomas, there are superficial spreading melanomas, lentigo maligna and desmoplastic melanomas. Non-solar malanomas are acral melanomas, melanomas in congenital nevi, melanomas in blue nevi, Spitz melanomas, mucosal melanomas and uveal melanomas. For epidemiology, clinical features, histopathology and differential diagnosis of each typology, please see the review by Elder and co-workers [5]. At the time of the diagnosis, the patients are generally treated by surgical excision of the primary tumor [2]. Unfortunately, very often patients develop metastases [7].

Melanoma is considered a multi-factorial disease, and the most well-known contributing factors are genetic susceptibility, familiar history and external stimuli, mainly sun exposure (due to its genotoxic effect) and a history of sunburn, as well as artificial UV exposure with tanning beds or psoralen-UVA radiation photochemotherapy [2,8,9,10]. The highest risk is often associated with histories of sunburn in childhood [11].

## 2. Marine Microorganisms

### 2.1. Bacteria 

In 2012, Yang and collaborators isolated 131 strains of actinomycetes from deep waters, collected from a depth of 800 m in Sagami Bay, Japan. They selected the AKA32 strain as a producer of cytotoxic compounds against murine cancer cells. They isolated three compounds from AKA32: the aromatic polychete akazamicin, actino-furanone C and N-formilan-tranilic acid. All three compounds showed cytotoxicity against the murine cell line of melanoma B16 with IC_50_ values of 1.7 μM, 1.2 μM and 25 μM, respectively [12]. In 2019, Schneider et al. [13], discovered that two bacterial isolates from the Barents Sea, belonging to the genus Algibacter, produced extracts with antibacterial and anticancer activity. They saw that both extracts had the same active ingredient identified as lipid 430. The effects of lipid 430 were tested against three human cell lines, melanoma A2058 cell line, HT29 colon cancer cell line and MRC5 lung fibroblast cell line. The compound was tested at concentrations of 233 μM, 175 μM, 116 μM, 58 μM, 23 μM and 12 μM. For the melanoma cell line a dose-dependent cytotoxic effect was observed, with IC_50_ 175 μM but there was no significant effect against the normal cell MRC5 [13]. In another work [14], anticancer and antimalarial assay were performed on a *Streptomyces* species (S.4) isolated from the marine sponge *Xestospongia muta* collected from Florida Keys. Active extracts from four *Streptomyces* isolates (S.1, S.2, S.3, S.4) were identified. The two extracts S.1 and S.2 have been found to have anti-proliferative activity with an IC_50_ of 2 µg/mL and 3.5 µg/mL, respectively, while the two extracts S.3 and S.4 showed antimalarial activity with an IC_50_ of between 2.5 µg/mL and 5 µg/mL for S.3 and an IC_50_ of 10 µg/mL for S.4. The S.3 extract showed both antiproliferative activity with an IC_50_ of 3.4 µg/mL and antimalarial activity with an IC_50_ of about 4 µg/mL. In particular, in the S.1 and S.2 extracts, the cytotoxic compounds nonactin, monactin, dynactin, and toyocamycin were found, and identified as responsible for the anti-proliferative activity. The compounds nonactin, monactin and dynactin were found to inhibit the proliferation of A2058 melanoma cells with IC_50_ of 0.26 µM, 0.02 µM and 0.02 µM, respectively, A2780 ovarian -cancer cells with IC_50_ of 0.2 µM, 0.02 µM and 0.02 µM, respectively, and H553-T non-small cell lung cancer cells with IC_50_ of 0.1 µM, 0.01 µM and 0.01 µM, respectively. Furthermore, the compounds monactin and dynactin showed some selectivity in melanomas; in fact, they were 6.5–13 times more active against the A2058 melanoma line than the A2780 ovarian cancer cell line [14]. Myxobacteria, has recently been recognized as a potential source of new secondary metabolites such as polyketides and ribosomal-free peptides, as well as their hybrid compounds [15,16]. Myxobacteria of marine origin are particularly attractive [17] because their gene sequences of polyketide synthase are unique. From a marine myxobacteria, *Enhygromyxa* sp. three new compounds were isolated: enigromic acid, deoxy-enigrolides A and deoxy-enigrolides B. Of these, enhygromic acid showed cytotoxicity against melanoma B16 cells with IC_50_ of 46 μM, comparable to that of the chemotherapy agent paclitaxel (57 μM), but it did not show activity against Hela-S3 cell (IC_50_ > 30 μM) [18]. 

Phenazine-1-carboxylic acid (PCA) has been produced, purified and characterized by the marine bacterium *Pseudomonas aeruginosa* GS-33 [19]. This compound showed a potent dose-dependent anticancer activity on SK-MEL-2 melanoma cells with a GI_50_ (growth inhibition of 50%) of 2.30 μg/mL (since a GI_50_ value of 10 μg/mL is considered to demonstrate anticancer activity in the case of pure compounds [20]). PCA has also been shown to have a protective effect against UV-B rays in evaluating its role in the enhancement of SPF (sun protection factor). The SPF of the PCA solution in ethanol at concentration 25 ppm, 50 ppm and 100 ppm were 1.43, 2.55 and 4.73, respectively. The addition of PCA (25 ppm, 50 ppm and 100 ppm) in the solution of two commercial sunscreens caused a synergistic increase of 10–30% in their SPF [19]. Two new lyso-ornithine lipids have recently been isolated from an arctic marine bacterium belonging to the genus *Lacinutrix* isolated from the sponge *Halichondria* sp. collected in the Barents Sea. The bacterial extract was fractionated into six fractions of which cytotoxic and antibacterial activities were tested at a concentration of 50 µg/mL. Fraction 5 was active against the Gram-positive bacteria *Streptococcus agalactiae*, *Enterococcus faecalis* and *Staphylococcus aureus*. Two lyso-ornithine lipids were found in this fraction. The cytotoxicity of these two lyso-ornithine lipids was evaluated against the human melanoma line A2058 at a concentration of 10 µM, 25 µM, 50 µM, 100 µM and 150 µM. A certain cytotoxic activity has been observed for one of the two lipids against the melanoma cell line A2058, with a cellular survival of 23% at 50 µM and a cell survival of about 0% at 100 µM and 150 µM, while the other lipid showed no activity against melanoma cells. The isolated compounds were tested on the normal lung-fibroblast MRC-5 cells and neither of them were active against normal cells [21].

### 2.2. Fungi

In 2014, Zhang et al. [22] isolated a derivative of sansalvamide A, the H-10, from the marine fungus belonging to the genus *Fusarum*. H-10 is a cyclic depsi-peptide that has shown a dose-dependent antiproliferative effect on B16 murine melanoma cells. The latter, treated with 50 µM of H-10, underwent morphological changes typical of the apoptotic process [22]. An alkaloid isolated in 2015, Penicitrinine A, from the marine fungus *Penicilium citrinum* was tested on A735 human malignant melanoma cells. Twenty-three tumor cell lines were treated with increasing concentrations of penicitrinine A for 48h, and the treatment showed inhibition of proliferation. The most sensitive cell lines were those of malignant melanoma A735 with an IC_50_ of 20.12 µM. They then evaluated with the Real-Time Cell Analysis (RTCA) test the inhibition of the specific proliferation of A735 and showed that this inhibition was related to the induction of apoptosis because, following treatment with 5 µM, 10 µM, 20 µM penicitrinine A, the cells began to shrink, round and fractionate, typical signs of apoptosis. The phenomenon was further confirmed by the staining test Annexin V-PI. The authors concluded that this alkaloid could favor the inhibition of the metastatic process in cancer cells [23].

Very recently, another compound Chlovalicin B was isolated from the marine fungus *Digiratispora marina*, taken from driftwood harvested in Vannoya in Norway in 2010 [24]. This compound exhibited mild cytotoxic activity against human A2058 melanoma cells with approximately 50% survival at 50 µM. No activity was observed against human normal lung fibroblasts MRC-5 at 50 µM, while mild activity was also seen in mouse melanoma cells B16 with an IC_50_ of 37 µM. The latter data may indicate that chlovalicins affect a common molecular target in melanoma cells [25]. In 2021, Jenssen et al. [26] discovered and isolated a new secondary metabolite, lulworthinone, from a slow-growing marine mushroom extract belonging to the Lulworthiaceae family. The compound was tested on A2058 melanoma cells, HepG2 hepatocellular carcinoma cells and normal lung fibroblast MRC-5 cells to evaluate its antiproliferative activity at concentrations ranging from 6.25 µg/mL to 100 µg/mL. The antiproliferative activity was observed against all cell lines tested. At concentrations of 20 µg/mL, 15 µg/mL, and 12.5 µg/mL the lulworthinone did not display toxic effect, with 100% cell survival. In the same year, Fan et al. [27], tested the fungal crude extract of *Pyrenochaetopsis* sp. FVE-001 on different tumor cell lines. This is an endophytic fungus isolated from thallus of brown seaweed *Fucus vesiculosus*. Three new compounds have been isolated from this fungus: pyrenosetin A, pyrenosetin B and pyrenosetin C, as well as a fourth compound already known, phomasetin. These three pyrenosetins show unique structures of decalinoylspyrotetramic acid characterized by a trans-decalinic ring, a spiro system fused with a carbonyl unit (cyclopentanone) and a terminal part of tetramic acid. The first two both showed antitumor activity, although pyrenosetin A had higher antitumor activity and lower cellular toxicity then pyrenosetin B. The third compound, pyrenosetin C, showed a low IC_50_ in A375 cells, being inactive [27]. The natural bioactive products with trans-decalinic ring are common in fungi (e.g., *Fusarium, Penicillium* and *Alternaria*) [28]. The crude extracts were tested at a concentration of 100 µg/mL on 5 human tumor cell lines: HT29, A374, A549, HCT116, MDA-MB231 in addition to the HaCaT immortalized human keratinocyte line used as a control. Regarding results on the human melanoma cell line A375, the pyrenosetic A had an antitumor activity with an IC_50_ of 2.8 µM, pyrenosetic B also showed an antitumor activity with an IC_50_ of 6.3 µM, while pyrenosetic C and phomasetin had lower IC_50_ values of 140.3 µM and 37.3 µM, respectively. Toxicity was evaluated on HaCaT cells, where they noted that the IC_50_ of pyrenosetic A, pyrenosetic C and phomasetin compounds on the normal cells, were similar to those of melanoma cells, indicating that the compounds are not selectively toxic. On the other hand, the pyrenosetic B showed a lower toxicity value on HaCaT with IC_50_ of 35.0 µM, indicating a slightly better selectivity than the other three metabolites of around 5.6 (value calculated by dividing the IC_50_ against HaCaT cells by the IC_50_ against melanoma cells A375).

### 2.3. Microalgae

Although the use of microalgae is very promising, in some cases a problem is that the rigid cell walls of microalgae need to be destroyed for the extraction of their bioactive compounds. Jabeen et al. [29], have evaluated the effect of enzymatic destruction of cell walls with cellulase and lysozyme, which was shown to be more advantageous than other conventional pre-treatment techniques, on the anti-tumor activity of microalgal extracts. They have evaluated the anticancer effect of the extract in the common cancer cell lines including the melanoma cell line MDA MB-435. The samples treated with lysozyme performed slightly better than cellulase-treatment on MDA MB-435 tumor cells [29]. However, other methods are also used for cell breakage, such as the use of sonication [30,31,32,33].

Oxylipins are metabolites derived from the lipid peroxidation [34]. The oxylipins 13-HOTE and 15-HEPE, derived from the microalga *Chlamydomonas debaryana* and *Nannochloropsis gaditana,* respectively, have been investigated for their activity on melanoma cancer cell line UACC-62. They showed high cytotoxicity on UACC-62 cells with IC_50_ values of 71.9 ± 3.6 μM for 13-HOTE and 53.9 ± 6.4 μM for 15-HEPE. In particular, the oxylipin treatment decreased the level of ATP in UACC-62 in a dose-dependent manner. These effects were magnified when oxylipins were combined with the glycolysis inhibitor 2-DG [35]. Lauritano and collaborators [30] found that raw extracts of the diatom *Skeletonema marinoi* (clone FE60) were active against A2058 melanoma cells when tested at 25–100 μg/mL. In particular, they cultivated the algae in replete medium and phosphate and nitrogen starvation, and found that only the pellets deriving from the nitrogen-starvation condition showed anti-melanoma activity, suggesting that in this condition the algae were able to produce, or produce more of, an amount of potential bioactive compound/s. At the same time, the nitrogen-starvation derived extracts were not toxic on normal human lung fibroblast MRC-5 or human hepatocellular liver carcinoma HepG2. Riccio et al. [31] also found activity against A2058 melanoma cells by raw extracts and fractions of the flagellate *Isochrysis galbana* cultured for 6 or 12 days, mainly at 100 μg/mL. However, some fractions also showed activity on MRC-5 cells.

The anticancer effect of the Amphidinol 22 isolated from the dinoflagellate *Amphidinium carterae* has been tested on the human skin melanoma cell line A2058. To test the antitumor activity, a MTT assay was conducted. The compound showed cytotoxicity with an IC_50_ of 16.4 µM [36]. Other *Amphidinium* spp. compounds have been previously reported to have an antitumor activity, such as the cytotoxic macrolides amphinolide G and amphinolide H. These two compounds exhibited extremely strong cytotoxic activities on KB human epidermoid carcinoma cells with IC_50_ values of 0.0059 and 0.00052 µg/mL, respectively [37]. In a work of 2019 [38], four new cytotoxic compounds have been characterized, three of them members of the macrolide amphidinolide family. Amphidinolides (AMPs) and related compounds are a diverse class of more than 40 macrolides with extremely high cytotoxicity against several carcinoma cell lines [39,40,41]. These were produced by symbiotic unicellular microalgae of the genus *Amphidinium*. The four new compounds, isolated from the invertebrate *Stragulum bicolor,* are: 5-membered macrolide amphidinolide PX1 (AMP-PX1), amphidinolide PX2 (AMP-PX2), amphidinolide PX3 (AMP-PX3) and the linear polyketide stragulin A. These compounds were tested between 8 µM to 8 nM against the A2058 cells derived from the metastatic site (lymphonode). Among these, the linear polyketide stragulin A was strongly and selectively active on the highly invasive melanoma cell lines A2058, with an IC_50_ of 0.18 µM after 48 h of treatment [38]. Water soluble polysaccharides have been isolated and purified from the biomass of the green alga *Parachlorella kessleri* HY1, and their immunomodulatory activities were evaluated on splenocytes from homogenized spleens of healthy and melanoma bearing C57Bl/6 mice. The polysaccharide tested with immuno-spot assay increased the production of INF-γ in the melanoma cells [42]. In another study, the sulpho-glycolipidic fraction of the red microalgae *Porphiridium cruentum* has been tested [43]. This fraction had large amounts of palmitic acid (26.1%), arachidonic acid (C20: 4ω-6, 36.8%), and eicopentaenoic (C20:5ω-3, 16.6%) acids, and noticeable amounts of 16:1n-9 fatty acid (10.5%). These could have a chemotherapeutic or chemoprotective potential, because they inhibited the growth of human malignant melanoma cells M4 Beu. They clearly showed a strong efficacy of the sulpho-glycolipidic fraction on all tested cell-lines, as demonstrated by IC_50_ values for growth inhibition in the range of 20–46 µg/mL. The sulpho-glycolipidic fraction inhibited growth-rates of both cytotoxic and cytostatic effects and blocked the cell cycle at a step corresponding to a transient increase of cell metabolism [43]. Another compound that showed anticancer activity on different human cutaneous melanoma cell lines is euplotin C, a secondary metabolite isolated from the marine ciliate *Euplotes crassus* [44]. At molecular levels, inhibition of ERK (extracellular signal-regulated kinase) and Akt (protein kinase B) pathway was shown to be induced in melanoma A375 cells by euplotin C. In particular, ERK 1/2 and Akt signaling pathways are often aberrantly activated in melanoma, inducing a complex network involved in melanoma cell proliferation and metastasis formation [44,45,46].

Euplotins are a group of compounds isolated from the marine ciliate *Euplotes crassus*. Subsequently, Carpi et al. [47] observed that euplotin C exerted cytotoxic effects on human melanoma cells A375, MeWo and 501Mel with an efficacy on these cells 30 times stronger than on normal cells’ HDF. Furthermore, euplotin C down-regulated the levels of B-Raf, ERK1/2 and p-Akt, promoting apoptosis by activating the ryanodine promoter (RyR) [48], and suppressed cell migration by inhibiting the ERK and AKT pathways [49]. Therefore, the authors suggested that euplotin C could be used in the treatment of melanoma as a selective activator of RyR, thus inducing apoptosis [47]. Finally, marine derived carbohydrates have potential skin health benefits. The skin barrier function of microalgae extract was assessed in anti-melanoma in vitro and in vivo studies [50]. These carbohydrates have been previously reported in the review by Kim et al. in 2018 [51]. 

Compounds with activity against melanoma isolated from bacteria, fungi and microalgae reported in the current review are summarized in Table 1.

## 3. Marine Macro-Organisms

Marine macro-organisms are a rich and precious source of anticancer active compounds. Many have been studied in several in vivo/in vitro/ex vivo experiments providing many compounds (listed in Table 2) with great in vitro/in vivo efficacy as anti-melanoma compounds. Each of them showed particular features as discussed below. 

### 3.1. Macroalgae

Spatane diterpenes from the marine brown alga *Stoechospermum marginatum* have been deeply investigated for their capability to selectively induce apoptosis in melanoma cells [53,54]. In more detail, spatane diterpenes induced apoptosis in in vitro experiments on melanoma murine cell lines [53,54] and also efficiently suppressed tumor development in vivo C57BL/6 mice engrafted with B16F10 melanoma cell line without apparent toxicity [54]. According to their findings, Spatane diterpenes stimulated the production of reactive oxygen species (ROS) leading to change in the Bax/Bcl-2 ratio and disruption of the inner mitochondrial transmembrane potential, cytochrome c redistribution, and activation of the caspase-mediated apoptotic pathway [54]. Moreover, they induced cell cycle arrest in “S-phase” and also caused apoptosis by disrupting the PI3K/AKT signaling pathway [54].

Fucoidan CF isolated from the alga *Chordaria flagelliformis* is a compound known to have anti-melanoma activity [55]. A combination of in vivo/ex vivo/in vitro experiments on murine animal model and melanoma cell lines elucidated the mechanism of action [55]. In particular, it has been demonstrated that Fucoidan CF stimulates the innate immune system via stimulation of CD11c integrins [55]. Fucoxanthin, found in the alga *Undaria pinnatifida*, showed specific in vitro cytotoxicity versus melanoma MALME-3M [56]. In vivo studies and further investigations are needed to explain the mechanism of action and validate the efficacy of this peculiar alga’s fucoxanthin as a candidate for melanoma therapy. Fucoxanthin derived from another alga, *Ishige okamurae*, has been used to unravel the molecular mechanisms of fucoxanthin’s protection, both in in vitro melanoma cell lines (B16F10 cells) and in vivo in Balb/c mice engrafted with B16F10 cells [51]. Apoptosis and cell cycle arrest during the G0/G1 phase were induced in B16F10 cells by fucoxanthin. Bcl-xL and IAP (inhibitor of apoptosis proteins) were down-regulated leading to the activation of caspase-9, caspase-3, and PARP [51]. Intraperitoneal fucoxanthin administration in Balb/c mice implanted with B16F10 cells considerably confirmed its in vivo anti-tumor efficacy [51]. Fucoxanthin (FX) derived from ethanol extracts of the brown alga *Fucus evanescens* was tested on human melanoma (SKMEL-28) cell lines [57]. Its antitumor efficacy was evaluated confirming inhibition in the growth of human melanoma cells perfectly in line with the previous above-mentioned studies [57]. One of the pharmacological effects of fucoxanthin is its anti-cancer action as an anti-metastatic action [58]. The anti-metastatic action of fucoxanthin, isolated from the brown alga *Saccharina japonica* has been demonstrated in in vitro experiments in B16F10 melanoma cell lines [58]. This effect could be due to the reduced expression of molecules involved in migration, invasion and adhesion: CD44, CXCR4 (CXC chemokine receptor-4) and MMP9 [58]. Fucoxanthin significantly reduced cell migration and decreased tumor nodules in experimental lung metastasis in an in vivo assay [58]. 

Two sulfated polysaccharide fractions (L.s.-1.0 and L.s.-P), obtained from the brown seaweed *Saccharina latissima*, were studied for possible activity against melanoma [59]. Mice subcutaneously inoculated with B16F10 cells were treated with both L.s.-1.0 and L.s.-P fraction. Hemoglobin content, the number of tumor-associated blood vessels, and tumor growth were significantly decreased, confirming the antiangiogenic and anticancer properties of these compounds [59]. In vitro studies analyzed the ability to prevent the proliferation of tumor cells of fucose-containing sulfated polysaccharides (FCSPs) from brown macroalgae *Sargassum henslowianum* (FSAR) and *Fucus vesiculosus* (FVES) to unravel the underlying apoptosis-inducing mechanisms [49]. Both FCSPs—FSAR and FVES—decreased the proliferation of melanoma cells and promoted apoptosis by FCSP’ mediated activation of caspase-3 [49]. Ale and colleagues also tested crude fucoidan isolated from *Sargassum* sp. (MTA) and *Fucus vesiculosus* (SIG) an in vivo melanoma murine model. They demonstrated that crude fucoidan increased natural killer cell activity in mice in vivo and had bioactive effects on melanoma model cells in vitro [60]. Polysaccharide fractions (SPPs), SPP-0.3, SPP-0.5, SPP-0.7, SPP-1, and SPP-2, purified from brown alga *Sargassum pallidum,* have been tested for their anticancer and immune-enhancing effects [61]. Chemical composition has been characterized using infrared spectroscopy [61] determining for each fraction the ratio of total saccharides, monosaccharide composition, and sulfated contents. Anti-tumor experiments showed that all SPPs lead to cancer cell death and have high anticancer activity against B16 melanoma cell lines [61]. SPP-0.7 was the most active against B16 cells (at 25 μg/mL) and as immune-enhancing fraction, and selected for further purification, which showed that it is a homogeneous polysaccharide. Its mechanism of action was further investigated showing that it can significantly induce cell apoptosis, cytokine secretion, and cellular stress response. It increased serum cytokines interleukin-6 and interleukin-1 beta, inducible nitric oxide synthase and tumor necrosis factor-α [61]. 

### 3.2. Sponges

Monanchocidin-A is a novel compound derived from sponges closely related to *Monanchora* species [62]. It has been tested in vitro using the NCI-60 Human Tumor Cell Lines Screen to investigate its potential anti-cancer activity. The NCI-60 screen provided 60 cell cancer lines to evaluate the dose-response created by a particular drug, thus comparing and selecting compounds that are most selectively for cancer lines (https://dtp.cancer.gov/discovery_development/nci-60/; accessed on 14 July 2022). The melanoma cell lines used for the screening were LOX IMVI, MALME-3M, M14, MDA-MB-435, SK-MEL-2, SK-MEL-28, SK-MEL-5, UACC257, and UACC-62 [62]. This research demonstrated Monanchocidin-A anticancer potential, indicating a peculiar activity against melanoma cell lines [62]. Further investigations are needed to understand the mechanism of action of this compound in melanoma cancer cells.

The anticancer properties of bengamides, sponge-derived natural chemicals that have been identified as inhibitors of methionine aminopeptidases (MetAPs), have been extensively studied for their anticancer activity [63,64,65]. The inhibition of methionine aminopeptidases (MetAPs) leads to cell cycle arrest [66]. Starting from this evidence, Wenzel and colleagues set up a method to produce, and enhance bengamides’ characteristics from the terrestrial myxobacterium *Myxococcus virescens* [16]. The efficacy of derived and modified versions of bengamides was tested in a murine animal model affected by an early stage B16 melanoma [16]. The greatest safe dose antitumor activity in vivo was 60 mg/kg [16]. The anti-melanoma activity was significant, but moderate when compared with Docetaxel, used as a reference to test in vivo efficacy [16]. Despite antitumor efficacy being limited, the approach proved the benefits of combining genetic engineering and synthetic techniques for the cost-effective manufacture of optimized bengamides [16]. 

Jaspine-B is a pro-apoptotic compound, isolated from the marine sponge *Jaspis* sp. extract, identified for its ability to selectively kill in vitro experiment murine B16 and human SK-Mel28 melanoma cells [67]. The pro-apoptotic mechanism of action of Jaspine-B was exerted via inhibition of sphingomyelin synthase with disruption in ceramide metabolism that in turn leads to cell death [67]. Ascophyllan sulfated polysaccharide from brown seaweed *Ascophyllum nodosum* [68] has been found to inhibit the migration and adhesion of B16 melanoma cells by reducing the expression of N-cadherin and enhancing the expression of E-cadherin [69]. The exerted mechanism of action is due to the inhibition of the expression of matrix metalloprotease-9 (MMP9), thus affecting its secretion and the extracellular matrix environment. This peculiar activity has been proved in the in vivo murine melanoma model B16, where treated animals showed significantly reduced metastasis compared to the control group [69].

Halichondrin-B, is a potent cytotoxin isolated in the 1980s from two marine sponges*: Halichondria okadai* and *Lissodendoryx* sp. [70], with great cytotoxicity in the B-16 melanoma cancer cell line. An analogue of Halicondrin-B, eribulin mesylate, has been FDA approved (as Halaven^®^) in 2010 for the treatment of patients with metastatic breast cancer who have previously received at least two chemotherapeutic regimens for the treatment of metastatic disease, and in 2016, for the treatment of inoperable liposarcoma for patients who received prior chemotherapy that contained an anthracycline drug (from https://techtransfer.cancer.gov/aboutttc/successstories/eribulin-mesylate; accessed on 3 August 2022).

Cytotoxic bioassays were performed on arenosclerins A-C and haliclona-cyclamine-E, two novel tetracyclic alkyl-piperidine alkaloids isolated from the marine sponge *Arenosclera brasiliensis* [71]. The above-mentioned alkaloids have been reported to have cytotoxic action against B16 melanoma cancer cell lines at doses ranging from 1.5 to 7.0 mg/mL, showing that they had significant melanoma toxic activity [71].

### 3.3. Mollusks, Cnidarians and Echinoderms

A group of marine compounds, belonging to the family of lamellar alkaloids, have been isolated from the mollusk *Lamellaria* sp. and found, for the first time, to induce cancer death [72]. Ballot et al. tested lamellarin D on HBL skin melanoma cells showing that this compound induced senescence by arresting them in the G2 phase of the cellular cycle. The growth arrest due to senescence, induced by lamellarin D, is due to its effect on DNA Topoisomerase I [73]. 

*Holothuria parva*, popularly known as the sea cucumber, is an important aquatic marine organism with a variety of active pharmacological compounds. Sea cucumber compounds have been proven to have anticancer properties via inducing the pro-apoptotic pathway [74]. One of the primary factors that contribute to drug resistance in melanoma is a deficiency in apoptosis [75]. The specific toxicity and apoptotic effect of three sea cucumber extracts at different concentrations (250, 500, and 1000 mg/mL) on skin mitochondria isolated from melanoma mice animal models were proved to both increase the formation of reactive oxygen species (ROS) and the release of cytochrome c from the mitochondria only in the melanoma group [74]. Further investigation is needed to identify the potentially bioactive chemicals discovered in *H. parva* to confirm the selective pro-apoptotic melanoma effects. Sarcophine, (+)-7,8-dihydroxydeepoxysarcophine and Sarcophytolide, natural compounds derived from the Red Sea soft coral *Sarcophyton glaucum*, were tested for their possible inhibitory effects on the growth of murine-derived melanoma B16F10 cells [76]. Sarcophine and (+)-7,8-dihydroxydeepoxysarcophine selectively reduced melanoma cell growth after 48 h and 72 h treatment at concentrations which did not show cytotoxicity on monkey kidney CV-1 cells. The proposed mechanism of action for these compounds is the inhibition of de novo DNA synthesis and the increased PARP activity leading to cell death [76]. These features give a potential role for these compounds as melanoma anticancer drugs [76]. 

### 3.4. Tunicates

Recently, the antimicrobial peptides turgencin-A and turgencin-B, as well as their oxidized counterparts, were isolated from the Arctic maritime colonial ascidian *Synoicum turgens* by Hansen and colleagues [77]. Turgencin-A showed stronger cytotoxicity activity than Turgenicin-B in melanoma cell line A2058 with IC_50_ of 1.4 μM [77]. Cytotoxic activity was evaluated using AqueousOne cytotoxic reagent (Promega, Madison, WI, USA) [77]. Ecteinascidin-743 (ET743) is a new antitumor agent derived from *Ecteinascidia turbinata*, a Caribbean tunicate [78]. It exhibits strong cytotoxic and antitumor properties due to its alkylating properties [79]. Jimeno and colleagues proved in vitro the specific DNA minor groove’s guanine-specific alkylating feature of ET743 [79]. The antitumor efficacy of ET743 was then assessed in human melanoma tumor xenografts. ET743 (0.1 mg/kg) was extremely active in the chemo-sensitive melanoma MEXF 989 and tumor regression was detected in the first week after the start of treatment [80]. Palmerolide-A was identified from the tunicate *Synoicum adareanum* isolated from the Antarctic area. It has been shown to inhibit V-ATPase resulting in strong and specific cytotoxicity on melanoma cell line UACC-66 [81]. Many years later (2020), Murray and colleagues investigated the *Synoicum adareanum* microbiome composition to increase knowledge of the palmerolide-A biosynthetic pathway [82] and opened a new perspective on this precious marine natural product (MNP). Further in vivo investigations are needed to confirm Palmerolide-A as a potential candidate for melanoma treatment.

Thiaplidiaquinones A and B, marine meroterpenoid alkaloids derived by *Aplidium conicum,* have been investigated for their anti-tumoral properties [83] and the mechanism of cell death has been elucidated [83]. The natural products were found to be modest inducers of ROS but the dioxo-thiazine regio-isomer of thiaplidiaquinone A and a synthetic precursor of thiaplidiaquinone B were discovered to be moderately powerful inducers of ROS [83]. In addition, in vitro experiments on NCI sub-panel selectivity for melanoma cell lines demonstrated that the synthetic dioxo-thiazine regio-isomer of thiaplidiaquinone A is more effective in inhibiting melanoma cell growth compared with their natural products [83], emphasizing the crucial role that natural product total synthesis may play in new drug discovery. Compounds with anti-melanoma activity from marine macro-organisms are summarized in Table 2.

**Table 2:** Marine macro-organism derived compounds or extracts with activity in vitro or in vivo against melanoma. Pre-clinical studies showing marine-derived compounds with anti-melanoma activity in vitro/in vivo, mechanism of action (when known), marine organisms and experimental conditions are reported for each compound. Extract (ex); N/A (Not Available); Inhibitory concentration of 50% (IC_50_); growth inhibition of 50% (IG_50_); Lethal Concentration (LC_50_); Phosphoinositide 3-kinase (PI3K); Protein-kinase B (Akt); C-X-C chemokine receptor type 4 (CXCR4); Matrix metallopeptidase 9 (MMP9); Poly ADP-ribose polymerase (PARP); Vacuolar-type ATPase (V-ATPase); every four days (q4d).

Compound	Marine Organism	In Vitro/In Vivo	IC50/GI50/LC50 or Tested Concentration	Administration	Mechanism of Action	Ref.
	Macroalgae					
Ascophyllan	*Ascophyllum Nodosum*	In vivo mel animal model B16	25 mg/kg	Intraperitoneal Injection	Inhibition of matrix metallo-protease-9	[69]
Spatane diterpinoids	*Stoechospermum marginatum*	In vitro on melanoma cell lines:B16F10 In vivo animal model C57BL/6 grafted with B16F10 melanoma cell line	IC_50_ 3.95 μM 4, 10, 15 mg/Kg	In cell culture media Intraperitoneal injection	Apoptosis via activation of the caspase-mediated apoptotic pathway and PI3K/Akt pathway	[54]
Fucoidan CF	*Chordaria flagelliformis*	In vivo/ex vivo murine model grafted with B16 melanoma cell line	0.01 mg/mouse	Intravenous injection	Stimulation of the innate immune system via CD11c integrins	[55]
Fucoxanthin containing extracts	*Undaria pinnatifida*	Melanoma cell line Malme-3M	IC_50_ (48 h) 27.96 ± 1.36 μM IC_50_ (72 h) 17.33 ± 2.65 μM	In cell culture media	N/A	[56]
Fucoxanthin (FX)	*Fucus evanescens*	Human melanoma SKMEL-28 cell line	IC_50_ 114 μM	In cell culture media	Inhibition of the growth of human cell melanoma	[57]
Fucoxanthin	*Ishige okamurae*	B16F10 melanoma cell line	30 μM	In cell culture media	CD44, CXCR4 and MMP9 reduction	[58]
L.s.-1.0 fr. (O-sulfated mannoglucuronofucans) L.s.-P fr. (sulfated polysaccharides)	*Saccharina latissima*	B6 mice inoculated with B16F10 melanoma cell line	50 mg/kg	Intraperitoneal injection	Anti-angiogenesis	[59]
FSAR(fucoidanfr) FVES(fucoidan fr) Crude Fucoidan	*Sargassum henslowianum* *Fucusvesiculosus*	B16 melanoma cell line C57BL/6JJCL mice	0.2–0.8 mg/mL 50 mg/kg body wt	In cell culture media In vivo injection	Apoptosis mediated by activation of caspase-3	[49,60]
Polysaccharide fractions (SPPs)	*Sargassum pallidum*	B16 melanoma cell line	25, 100, and 400 μg/mL	In cell culture media	immune stimulation	[61]
	Sponges					
Monanchocidin-A	*Monanchora* sp.	In vitro on melanoma cell lines: -LOX IMVI -MALME-3M -M14 -MDA-MB435 -SK-MEL-2 -SK-MEL-28 -SK-MEL-5 -UACC257 -UACC-62	GI_50_ 0.022 μM GI_50_ 0.095 μM GI_50_ 0.018 μM GI_50_ 0.023 μM GI_50_ 0.13 μM GI_50_ 0.063 μM GI_50_ 0.034 μM GI_50_ 0.035 μM GI_50_ 0.024 μM	In cell culture media	N/A	[62]
Bengamides	*Myxococcus virescens*	B16 melanoma murine model	60 mg/kg	Mice injection	Inhibition of methionine amino peptidases [66]	[16]
Jaspine-B	*Jaspis* sp.	In vitro on melanoma cell lines: Human SK-Mel28; Murine B16	IC_50_ 0.5 μM	In cell culture media	Cell death via inhibition of sphingomyelin synthase	[67]
Halichondrin B	*Halicondria okadai* *Lissodendoryx* sp.	In vitro on B-16 melanoma cancer cells	IC_50_ 0.09 ng/mL	In cell culture media	N/A	[70]
Arenosclerin-A Arenosclerin-C Haliclonacyclamine E	*Arenosclera brasiliensis*	In vitro on B16 melanoma cell line	1.5–7.0 mg/mL	In cell culture media	N/A	[71]
	Mollusks, Cnidarians and Ehinoderms					
Lamellarin D	*Lamellaria* sp.	HBL skin melanoma cells	5 μM	In cell culture media	Arresting cells in the G2 phase of the cellular cycle due to its effect on DNA Topoisomerase I	[73]
Metanolic, ex Diethyl ether ex n-hexane ex	*Holothuria parva*	In vitro/Ex vivo	250, 500, and 1000 μg/mL	In cell culture media	Pro-apoptotic	[74]
Sarcophine (+)-7α,8β dihydroxydeepoxysarcophine	*Sarcophyton glaucum*	B16F10 melanoma cell line	500 μM	In cell culture media	Inhibit DNA synthesis and PARP activity	[76]
	Tunicates					
Turgencin-A	*Synoicum turgens*	In vitro on melanoma cell lines: A2058	IC50 1.4 μM	In cell culture media	N/A	[77]
Ecteinascidin-74	*Ecteinascidia turbinata*	Ex vivo	q4d x 3—0.2, 0.1, 0.05 mg/kg	Intravenous	Double-strand breaks (DBSs) [84,85]	[80]
Palmerolide-A	*Synoicum adareanum*	In vitro on melanoma cell line: UACC-66	LC_50_ 0.018 μM	In cell culture media	Inhibition of V-ATPase	[81]
Thiaplidiaquinones A and B	*Aplidium conicum*	In Vitro on NCI panel	10 μM	In cell culture media	Pro-apoptosis	[83]

## 4. Prevention of Damage Induced by UV Solar Radiation

**Figure 2:**
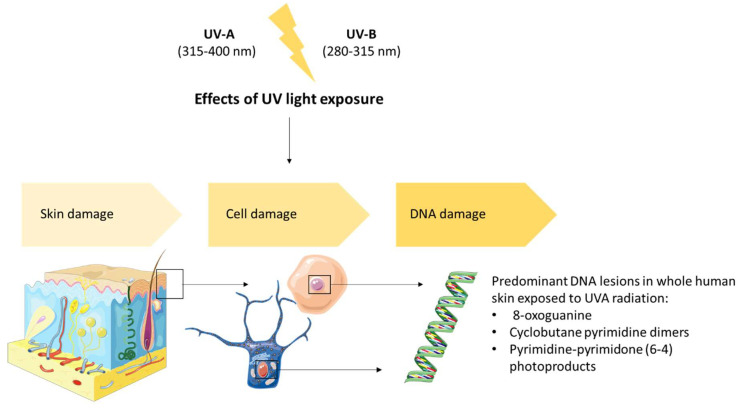
Effects of UV light exposure.

Inflammation induced by UVB rays and the formation of reactive oxygen species (ROS) are involved in the development of melanoma; in fact, UV radiation is an environmental carcinogen that in high doses can cause damage to the skin and induce cancer [5] (Figure 2). UVB increases the cutaneous activity of ornithine decarboxylase (ODC), the first enzyme in the polyamine biosynthesis pathway. This may cause excessive proliferation and clonal expansion of the cells initiated, leading to tumorigenesis [86,87].

**Figure 3:**
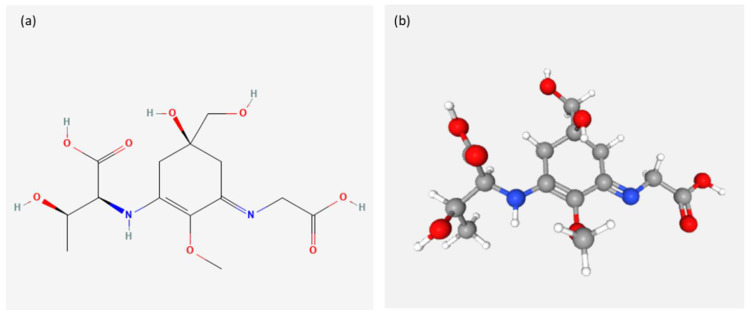
Chemical structure (**a**) 2D and (**b**) 3D of Porphyra-334 (PubChem Identifier: CID 6857486) from https://pubchem.ncbi.nlm.nih.gov/compound/Porphyra-334#section=2D-Structure&fullscreen=true and https://pubchem.ncbi.nlm.nih.gov/compound/Porphyra-334#section=3D-Conformer&fullscreen=true, respectively (accessed on 13 July 2022) [100].

Marine organisms have developed a wide variety of adaptive strategies to obviate the effects of UV radiation and the best known photoprotective response is the production or accumulation of compounds that absorb UV. Among these compounds are myco-sporine-like amino acids (MAA), scytonemin, 3-hydroxyquinurenine, melanin, various secondary metabolites and fluorescent pigments [83,84,85]. The MAAs are commonly known as ‘‘microbial sunscreens’’ [88,89]. MAAs have the ability to absorb light between 309 and 362 nm by dissipating radiation in the form of heat without producing reactive oxygen species (ROS) [90]. MAAs have been found in a large variety of marine organisms, including bacteria, cyanobacteria [91,92], fungi [93] and microalgae [94].The MAA content varies seasonally, peaking in the summer, in the various organisms [95]. They have many advantages, as they protect cells from mutations caused by UVR rays and free radicals and are effective antioxidant molecules [92]. Thanks to their multiple roles, MAAs are well regarded for applications in the pharmaceutical and cosmetic industries as natural sunscreens, cell proliferation activators, anticancer agents, anti-photoaging molecules and skin renewal stimulators [96]. An example of a product containing MAA and marketed as Helioguard^®^ 365 sunscreen, is porphyra-334 from the red alga *Porphyra umbilicalis* associated with shinorine, which has protective properties against the loss of cellular vitality and DNA damage induced by UVA rays [97,98]. Helionori^®^ sunscreen is another product containing MAAs, palitin, porphyria-334 (Figure 3) and shinorine as active ingredients, extracted from *Porphyra umbilicalis*, which protects from UV-A rays, preserving the membrane lipids of keratinocytes and fibroblasts, in addition to DNA protection [98,99].

Scytonemin is a pigment produced mainly by cyanobacteria [101,102]; thanks to its multiple roles as UV sunscreen and antioxidant with strong radical scavenging activity, it is a very interesting natural product for the formulation of sunscreens destined for the market [103,104]. It also exhibits antiproliferative and anti-inflammatory activities in human fibroblasts and endothelial cells [101,105,106]. Scytonemin inhibits a serine/threonine kinase, named Polo-like Kinase 1, which plays a key role in regulating the G2/M transition in the cell cycle [106]. Carotenoids are also excellent allies for the prevention of diseases due to UV solar radiation and have applications in the healthcare and nutraceutical industry, for skin protection, anti-aging and as sunscreens, as they are powerful antioxidants and scavenging agents [107,108,109]. Microalgae are known as a valuable source of carotenoids [110]. An example of the most innovative skin care products from microalgae is Dermochlorella^®^ by CODIF Recherche et Nature (Brittany, France), an extract from the green microalgae *Chlorella vulgaris* containing oligopeptides that increase skin firmness and tone (http://www.codif-tn.com/en?s=dermochlorella; accessed on 11 July 2022) [109].

Among the various pigments currently used in cosmetics produced by marine organisms, such as macro and microalgae, there is fucoxanthin (FX) which is able to counteract the oxidative stress caused by UVR [87,98,111,112,113]. Its photoprotective action is more effective when it is used in topical preparation [87]. For example, UV solar radiation exposure can cause hyper-pigmentary disturbances (HD). A common example of HD are freckles, which are real skin lesions and indicators of risk for skin cancer (melanoma and non-melanoma). HDs are the consequence of increased production of pro-melanogenic factors and altered expression or activity of melanocyte receptors [87,114]. There are many studies showing that FX is an excellent candidate for the treatment and prevention of HDs. In guinea pigs irradiated for 14 days with incremental UVB doses, FX applied after UVB irradiation in form of food (10 mg/kg) or ointment (50 µL of white petrolatum containing 0.01–1% of FX) blocked cellular melanogenesis for six to ten days after the last irradiation session [115]. Another work showed that the application of a 0.5% FX Vaseline-based cream on day five after four days of UVB chronic irradiation (1 h per day, 2.7 J/cm^2^) on female ddY strain mice efficiently cured the sunburn [116]. A 2020 study showed that FX enhanced the antioxidant properties of a standard sunscreen containing avobenzone and ethylhexyl methoxycinnamate in a reconstructed skin model [117].

α-tocopherol is the most biologically active form of vitamin E, found in the thylakoid membranes of photosynthetic organisms, where it counteracts the effects of ROS by removing oxidized substrates or by blocking the lipid peroxidation chains initiated by ROS [118]. α-tocopherol has been shown to reduce inflammation and act as an antioxidant by reducing UV and ROS-induced damage in human and mouse skin cells [119,120,121,122,123]. α-tocopherol is produced by many marine organisms: it has been found in the microalga *Dunaliella salina* (where it represented 37.5–46.9 mg/100 g dry weight) [124], in *Chondrus yendoi* (9.34 mg/100 g), *Sargasso fusiforme* (3.56 mg/100g) and *Sargassum horneri* (3.65 mg/100 g) [125].

**Figure 4:**
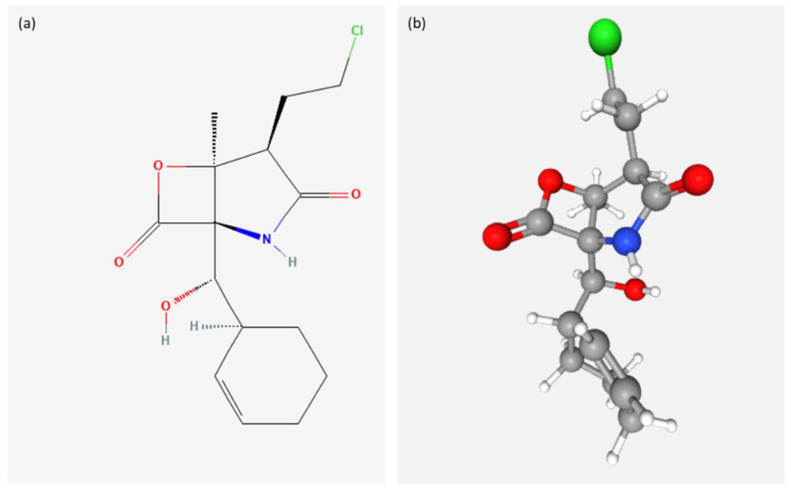
Chemical structure (**a**) 2D and (**b**) 3D of Marizomib (PubChem Identifier: CID 11347535) from https://pubchem.ncbi.nlm.nih.gov/compound/11347535#section=2D-Structure&fullscreen=true and https://pubchem.ncbi.nlm.nih.gov/compound/11347535#section=3D-Conformer&fullscreen=true, respectively (accessed on 13 July 2022) [135].

The application of marine natural products has been shown to be effective in reducing inflammation and oxidative stress [120]. For example, natural products such as 5β-scymnol and CO(2)-supercritical fluid extract (CO(2)-SFE) of mussel oil contain antioxidant and anti-inflammatory properties and they can help reduce the harmful effects of UV solar radiation [126]. In fact, a study was conducted to evaluate the anti-inflammatory effect of these compounds on normal cells derived from human epidermal melanocytes (HEM) in relation to α-tocopherol. HEM cells were irradiated with UVB and treated with IL-1 alpha. When α-tocopherol, CO(2)-SFE mussel oil, and 5β-scymnol were added, TNF-α levels decreased, respectively, by 53%, 65% and 76%, which was not observed in malignant melanoma cells MM96L. The pro-inflammatory cytokine TNF-α has been shown to be involved in the progression of melanoma through the inhibition of apoptosis [127,128]. Therefore, these compounds can be used in the prevention of inflammation-induced damage of normal melanocytes. Both UVA and UVB can trigger oxidative responses that may persist after the end of exposure to UV radiation sources [129]. DNA oxidative damage caused by melanin sensibility to UVA radiation is involved in melanogenesis [130] (Figure 4). UV radiation is known to trigger multiple signaling cascades such as mitogen-activated protein kinase P38 (MAPK), terminal kinase c-Jun (JNK), extracellular kinase regulated by signal 1/2 (ERK1/2) and nuclear factor pathways κB (NFκB) in the skin cells [126,131,132,133]. A strategy to mediate the effects of UV radiation on the skin can act on these pathways. As reported by Sample and He [134], research studies have shown that sunscreen is often ineffective at reducing melanoma risk; hence, melanoma prevention can be improved by further research and trials of sunscreen products, as well as optimization of their design.

## 5. Discussion

Malignant melanoma is among the most dangerous tumors due to its high probability of metastasizing and its increasing incidence year after year [136]. Currently, 75% of skin cancer deaths are due to melanoma [137]. There are three types of skin tumors: Melanoma, Basal Cell Carcinoma (BCC) and Squamous Cell Carcinoma (SCC). BCC and SCC are not fatal and can be treated surgically. Melanoma skin cancer develops when the melanocytes (cells that normally make melanin pigment) start to grow out of control. Melanomas are fatal and the victims are eight times greater in number than those with non-melanoma skin cancers, because it is much more likely to spread to other parts of the body if not treated early. Melanomas are etiologically divided into melanomas related to sun exposure and those which are not, but also based on their mutational signatures, anatomic site, and epidemiology [138]. Bobos, in a review of 2021, gives an overview of the latest news concerning the histopathologic classification of various types of skin cancer [139]. What is similar between the various types of melanoma is the final stage of development which consists in the formation of local and/or distant metastases [139].

Understanding more deeply the molecular mechanism of action that leads to the onset of melanoma may allow the identification of possible molecular targets. There are already eight molecular subtypes of melanoma identified [140], thanks to the study of the different types of molecular anomalies. Knowing the molecular mechanism underlying the onset of melanoma can also make it easier to identify and discriminate the natural substances that can act in a specific way on these molecular targets, which implies the possibility of developing targeted therapies.

Prolonged and incorrect exposure to UV rays is one of the main causes of the onset of melanoma. Sun exposure without sunscreen, sun exposure in the hottest hours, sunburn and underestimating the harmfulness of UV rays, even when it is cloudy, are behaviors that can lead to an increased risk of skin cancer. Not everyone is genetically predisposed to tan; this is due to the presence of two different types of melanin which are expressed with varying percentages in each individual [141]. A darker complexion is characterized by increased production of the eumelanin pigment (brown/black) which gives a brown color and protects against UV damage [141]. A fair complexion is determined by the increased production of the pheomelanin pigment (red/yellow) which is responsible for the redness of the skin and does not protect from UV rays [141]. For this reason, individuals with fair complexions are more prone to skin cancers than individuals with a dark complexion, but this does not exclude that latter, who are not immune from damages caused by UV rays. In some countries, there is a misconception that more tanned or colored skin is a sign of good health and beauty [142]. It is therefore essential also to focus on the production of creams with specific SPFs for each skin type, suitable for skin protection and the prevention of skin tumors.

Melanoma has also been found in marine species. For instance, Sweet and co-workers [143] found melanosis and melanoma in wild populations of the coral trout *Plectropomus leopardus*, which is a commercially important marine fish. The presence of melanoma not only in humans suggests new potential market sectors for compounds with anti-melanoma activity, not only for human application but also, for instance, for the aquaculture sector.

Marine organisms are a rich source of bioactive compounds that have been shown to exert various bioactivities, including anticancer, anti-inflammatory and immunomodulatory properties. To date, there are 14 marine derived drugs on the market, and several in clinical trials I, II and III, having great potential to increase the number of natural marine products in clinical use [136]. Among these, Marizomib (Salinosporamide A; NPI-0052) is currently in clinical trial III for melanoma treatment. It is a beta-lactone-gamma lactam, first isolated from a marine bacterium of the genus Salinospora [144] (https://www.midwestern.edu/departments/marinepharmacology/clinical-pipeline; accessed on 13 July 2022). The molecular target of Salinosporamide A (Figure 4) is 20S proteasome. Millward et al. [145] tested Marizomib, with or without combination with vorinostat on low metastatic cell lines (including SB2, DM4 and TXM13), intermediate metastatic cell lines (including Mel526, Me1624, Me1888, Me1938 and MeWo) and highly metastatic cell lines (including WM2664, WM293, WM793, WM35, A375SM, A375 and C8161). They observed that the combination Marizomib and vorinostat had the strongest activity on highly metastatic melanoma cell lines. In the current review, we report compounds deriving from marine micro- and macro-organisms with activity on melanoma cells. The most active, considering the lowest active concentrations, are Actinofuranone C from AKA32 strain of actinomycetes *Nonomuraea* sp. with an IC_50_ of 1.2 μM and Monanchocidin-A, isolated from the sponge *Monanchora* sp. with activity on M14 melanoma cell line with GI_50_ of 0.018 μM.

Considering the increasing market demand for new drugs against drug-resistant pathologies, and the search for compounds with reduced side effects, the attention of researchers is increasingly focused on natural substances and/or modification/conjugation of natural lead compounds in order to direct specific cell lines and cellular targets. According to the database MarinLit (https://marinlit.rsc.org/; accessed on 3 August 2022), which is specifically dedicated to marine natural products research, there are actually 38,990 marine compounds and about 38,713 published articles. According to the World Register of Marine Species (WORMS; https://www.marinespcies.org/news.php?p=show&id=4099, accessed on 3 August 2022), currently 228,450 species are known and every day new species are discovered and described. In addition to great biodiversity in terms of species, the oceans are characterized by huge chemical diversity and it was shown that approximately 70% of structural scaffolds identified at sea are only found in marine organisms, without any terrestrial counterpart [146,147]. Extreme environments, such as deep and cold, are less explored compared to more accessible sites and worth further investigation for new species and chemicals [148]. Marine microorganisms, being easy to handle, are considered an eco-sustainable and eco-friendly source of bioactive compounds for marine biotechnology [149]. In fact, almost 60% of new marine natural products today derive from microorganisms [2,150]. Marine microorganisms have also attracted great attention because they have developed metabolic and physiological capacities that guarantee their survival in extreme habitats and offer the potential to produce compounds with possible pharmacological activity [151,152]. In addition, for cultivable microorganisms, such as fungi, bacteria and microalgae, there also is the possibility of inducing the production of bioactive compounds by applying stressful exposure, such as changing culturing parameters (light, nutrient, temperature and others). This approach, known as “one strain–many compounds” or OSMAC, allows easier identification of new bioactive molecules [153]. For this reason, strategies to increase the probability of discovering new bioactive compounds, consist in searching less explored places [154,155,156], such as deep and cold waters, or focusing on cultivable species and inducing the production of other metabolites. Overall, the data reported in this review show that marine organisms may produce various chemical structures with activities against different melanoma cell lines, but also in in vivo models. The molecular mechanisms activated can be variable, ranging from immune-activation to apoptosis induction. In addition, for several compounds the mechanism of action is not completely clarified yet and, hence, are worth additional investigation in order to proceed with clinical trials.

## Figures and Tables

**Table 1:** Marine microorganism derived compounds or extracts with activity in vitro or in vivo against melanoma. Pre-clinical studies showing marine-derived compounds with anti-melanoma activity in vitro/in vivo, mechanism of action (when known), marine organisms and experimental conditions are reported for each compound. Inhibitory concentration of 50% (IC_50_); growth inhibition of 50% (IG_50_); extracellular signal-regulated protein kinase (ERK1/2); Phosphorylated protein-kinase B (p-Akt); adenosine triphosphate (ATP); Ryanodine promoter (RyR); Not available (N/A); B-cell lymphoma 2 (Bcl-2); bcl-2-like protein 4 (Bax).

Compound	Marine Organism	In Vitro/In Vivo	IC50/GI50/LC50 or Tested Concentration	Administration	Mechanism of Action	Ref.
	Bacteria					
Aromatic polychete akazamicin Actinofuranone C N-formilantranilic acid	AKA32 strain of actinomycetes *Nonomuraea* sp.	In vitro on melanoma cell B16	IC_50_ 1.7 μM IC_50_ 1.2 μM IC_50_ 25 μM,	In cell-culture media	N/A	[12]
Lipid 430	Genus *Algibacter*	In vitro on melanoma cell A2058	IC_50_ 175 μM	In cell-culture media	Inhibition of cell proliferation	[13]
Enigromic acid Deoxyenigrolides A Deoxyenigrolides B	Mixobacteria *Enhygromyxa* sp.	In vitro on melanoma cell B16	IC_50_ 46 μM	In cell-culture media	N/A	[18]
Phenazine-1-carboxylic acid (PCA)	*Pseudomonas aeruginosa* GS-33.	In vitro SK-MEL-2 melanoma cells	GI_50_ of 2.30 μg/mL since GI_50_ value of 10 μg/mL	In cell-culture media	Reduced cell density Induction of apoptosis	[19]
Lyso-ornithine lipids	Genus Lacinutrix	In vitro on melanoma cells A2058	50 µM, 100 µM, 150 µM	In cell-culture media	N/A	[21]
	Fungi					
H-10	Genus *Fusarum*	In vitro in melanoma model H10	50 µM	In cell-culture media	Induction of the apoptosis of cells via a mitochondrial pathway. Increased activity of caspases 3. Inhibition of cell growth.	[22]
Penicitrinine A	*Penicilium citrinum*	In vitro on melanoma cells A735	IC_50_ 20.12 µM	In cell-culture media	Induction of apoptosis by decreasing of the expression of Bcl-2 and increasing of the expression of Bax. Anti-metastatic effects. Inhibition of proliferation	[23]
Chlovalicin B	*Digiratispora marina*	In vitro on melanoma cells A2058	IC_50_ 37 µM	In cell-culture media	N/A	[24]
Lulworthinone	Lulworthiaceae family	In vitro on melanoma cells A2058	From 6.25 µg/mL to 100 µg/mL	In cell-culture media	Inhibition of cell proliferation.	[25]
Pyrenosetin A Pyrenosetin B Pyrenosetin C Phomasetin	crude extract of *Pyrenochaetopsis* sp. FVE-001	In vitro on melanoma cells A375	IC_50_ 2.8 µM IC_50_ 6.3 µM IC_50_ 140.3 µM IC_50_ 37.3 µM.	In cell-culture media	N/A	[27]
	Microalgae					
Oxylipin 13-HOTE	*Chlamydomonas debaryana*	In vitro on melanoma cancer cell line UACC-62	IC_50_ 71.9 ± 3.6 μM	In cell-culture media	Decreased the level of ATP in UACC-62 in dose-dependent manner	[52]
Oxylipin 15-HEPE	*Nannochloropsis gaditana*	In vitro on mela-noma cancer cell line UACC-62	IC_50_ 53.9 ± 6.4 μM	In cell-culture media	Decreased the level of ATP in UACC-62 in dose-dependent manner	[52]
Raw extracts	*Skeletonema marinoi* (clone FE60)	In vitro on melanoma A2058 cells	25-100 μg/mL	In cell-culture media	N/A	[30]
Raw extracts and fractions	*Isochrysis galbana*	In vitro on melanoma A2058 cells	100 μg/mL	In cell-culture media	N/A	[31]
Amphidinol 22	*Amphidinium carterae*	In vitro on melanoma cells A2058	IC_50_ 16.4 μM	In cell-culture media	N/A	[36]
Linear polyketide stragulin A	*genus Amphidinium/Stragulum bicolor*	In vitro on melanoma cell A2058 derived from metastatic site.	IC_50_ 0.18 µM	In cell-culture media	N/A	[37]
Euplotin C	*Euplotes crassus*	In vitro on melanoma cells A2058	N/A	In the cell-culture media	Down-regulation of the levels of B-Raf, ERK1/2 and p-Akt, promotion of the apoptosis by activation of the RyR	[44]

## Data Availability

Not applicable.
